# Microbiome recovery in adult females with uncomplicated urinary tract infections in a randomised phase 2A trial of the novel antibiotic gepotidacin (GSK140944)

**DOI:** 10.1186/s12866-021-02245-8

**Published:** 2021-06-15

**Authors:** Andrea Nuzzo, Stephanie Van Horn, Christopher Traini, Caroline R. Perry, Etienne F. Dumont, Nicole E. Scangarella-Oman, David F. Gardiner, James R. Brown

**Affiliations:** 1 Human Genetics, GlaxoSmithKline R&D, Medicines Research Centre, Gunnels Wood Road, Stevenage, SG1 2NY UK; 2 Functional Genomics, GlaxoSmithKline R&D, Collegeville, PA USA; 3 Development, GlaxoSmithKline R&D, Collegeville, PA USA; 4 Medicines Opportunities Research Unit, GlaxoSmithKline R&D, Collegeville, PA USA; 5 Human Genetics, GlaxoSmithKline R&D, Collegeville, PA USA; 6 Present Address: Kaleido Biosciences, 65 Hayden Avenue, Lexington, MA 02421 USA

**Keywords:** Gepotidacin, Microbiome, Antibiotic, Clinical trial, Urinary tract infection

## Abstract

**Background:**

With increasing concerns about the impact of frequent antibiotic usage on the human microbiome, it is important to characterize the potential for such effects in early antibiotic drug development clinical trials. In a randomised Phase 2a clinical trial study that evaluated the pharmacokinetics of repeated oral doses of gepotidacin, a first-in-chemical-class triazaacenaphthylene antibiotic with a distinct mechanism of action, in adult females with uncomplicated urinary tract infections for gepotidacin (GSK2140944) we evaluated the potential changes in microbiome composition across multiple time points and body-sites (ClinicalTrials.gov: NCT03568942).

**Results:**

Samples of gastrointestinal tract (GIT), pharyngeal cavity and vaginal microbiota were collected with consent from 22 patients at three time points relative to the gepotidacin dosing regimen; Day 1 (pre-dose), Day 5 (end of dosing) and Follow-up (Day 28 ± 3 days). Microbiota composition was determined by DNA sequencing of 16S rRNA gene variable region 4 amplicons. By Day 5, significant changes were observed in the microbiome diversity relative to pre-dose across the tested body-sites. However, by the Follow-up visit, microbiome diversity changes were reverted to compositions comparable to Day 1. The greatest range of microbiome changes by body-site were GIT followed by the pharyngeal cavity then vagina. In Follow-up visit samples we found no statistically significant occurrences of pathogenic taxa.

**Conclusion:**

Our findings suggest that gepotidacin alteration of the human microbiome after 5 days of dosing is temporary and rebound to pre-dosing states is evident within the first month post-treatment. We recommend that future antibiotic drug trials include similar exploratory investigations into the duration and context of microbiome modification and recovery.

**Trial registration:**

NCT03568942. Registered 26 June 2018.

**Supplementary Information:**

The online version contains supplementary material available at 10.1186/s12866-021-02245-8.

## Background

Gepotidacin (GSK2140944) is a novel first-in-class triazaacenaphthylene antibiotic that selectively inhibits type IIA topoisomerases (DNA gyrase and topoisomerase IV) through a previously unexploited mechanism which is distinct from existing fluorquinolone inhibitors of this complex [1]. Phase 1 and 2 clinical studies show that gepotidacin is well-tolerated and has demonstrated efficacy in patients with acute bacterial skin infections [2, 3] and uncomplicated urogenital gonorrhea caused by *Neisseria gonorrhoeae* [4].

An increasing area of clinical interest is the potential effects that antibiotics have on the composition of endogenous human microbiota and its pan-genome, the microbiome. One specific concern is the impact of antibiotics on increased risk of severe secondary infections such as recurrent *Clostridium difficile* [5]. Epidemiological studies have also associated frequent usage of oral antibiotics to increased risk for certain chronic diseases such as inflammatory bowel disease [6] and celiac disease [7]. Given the critical role of the microbiota in maintaining immune homeostasis, the impact of pharmacologic agents is an emerging consideration in patient care [8]. Although antibiotic effects on the microbiome have been well-studied in animal models [9], healthy volunteers under antibiotics regimes [10] and retrospective analyses of patient cohorts [11], to the best of our knowledge, few studies have measured the effects of antibiotics on the human microbiome and its recovery in patients with bacterial infections, as typically enrolled in Phase 2 or 3 clinical trials.

In support of the clinical development of gepotidacin, we report on the spatial and longitudinal changes in the microbiome as an exploratory endpoint in a Phase 2a single-center, open label clinical study that evaluated the pharmacokinetics of repeated oral doses of gepotidacin in adult females with uncomplicated urinary tract infection (uUTI) [12]. Our results suggest that gepotidacin is associated with temporary, yet significant reduction in patients’ microbiome diversity by end of its dosing 5 day regimen. However, there is significant rebound or recovery of the microbiota to near baseline levels within a 4-week period post-treatment which suggests that gepotidacin related effects on the three body sites’ microbiome are temporary and transient.

## Results

### Study design and sample collection

The microbiome analysis was an exploratory endpoint in a Phase 2A clinical trial (ClinicalTrials.gov: NCT03568942). Study design, protocols and primary findings for this trial were previously reported [12] (see Methods). Samples for microbiome analyses were collected from 22 subjects with informed written consent in accordance with study protocols at three time-points: Day 1 (pre-dose); Day 5 (end of dosing or post-dose) and; Follow-up (visit around Day 28 ± 3 days). Three different body sites were sampled, namely the gastro-intestinal tract or GIT (stool sample), pharyngeal cavity (saliva sterile swab) and vagina (vaginal sterile swab). A total of 156 samples were collected with consent from 22 individuals (Additional file 1: Supplementary Table S1). Microbiota composition was determined by Illumina miSeq DNA sequencing of 16S rRNA gene variable region 4 amplicons along with the appropriate positive and negative experimental controls. After strigent quality control evaluation, 141 samples were used for subsequent microbiome analyses (see Methods for complete laboratory and data analysis protocols). Overall DNA read quality was high with average sequencing depth of 132 ± 69 K reads per sample. The relative abundances of assigned bacterial taxa for each body site and time-point are given in Additional file 2: Supplementary Data File S1.

### Microbiota relative abundance and diversity

**Fig. 1:**
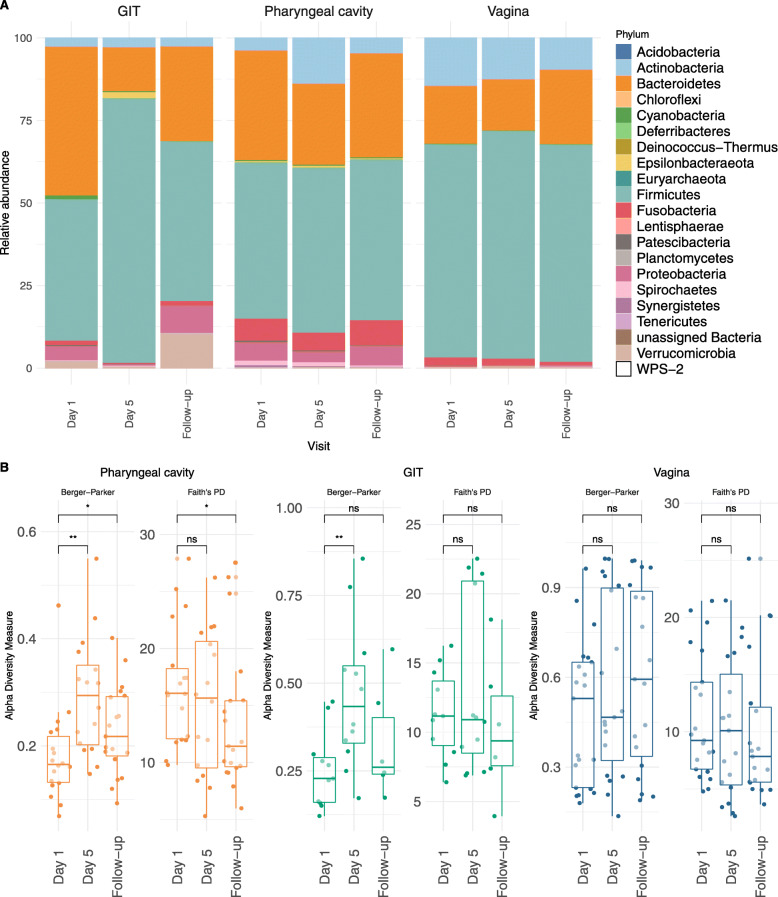
Overview of microbiome dynamics during gepotidacin Phase 2a clinical trial. (**A**) Phylum level changes in the relative abundance of microbiota across different body sites and time points. (**B**) Changes in microbiota community as measured by different indices of alpha diversity. The initial, lower placed value in each comparison is from the overall ANOVA (* *P* value ≤0.05; ** *P* value ≤0.005, ns = nonsignificant)

The three sampled body sites showed time-point related changes at the phylum level, represented for clarity as relative abundance in Fig. 1a. The greatest changes relative to time-points were observed for the pharyngeal cavity followed by the GIT and, finally, vagina. Proteobacteria, the predominant phylum of bacteria known to cause UTIs [13], was detected across all body sites at Day 1 with the greatest depletion occurring in GIT samples at Day 5.

Changes within microbial communities were measured by comparing different diversity indexes which gave comparable results (Fig. 1b and Fig. S1). Focusing on the Berger-Parker diversity index, the pharyngeal cavity showed notable declines in alpha-diversity (False Discovery Rate [FDR]-adjusted *P* value ≤0.05) at Day 5 and Follow-up relative to Day 1 but with a slight rebound trend at Follow-up. GIT microbiome showed a significant decline in alpha-diversity at Day 5 relevant to Day 1. Alpha-diversity values in GIT samples at Follow-up visit rose to levels that were non-significantly different from Day 1. In vaginal samples, alpha-diversity values trended lower at Day 5 and then higher at Follow-up with non-significant changes across all time-points. Similar conclusions could be drawn using different alpha diversity measures (Additional file 3: Supplementary Fig. S1a). An attempt to perform pairwise analysis resulted in loss of significant differences for GIT, but mainly due to patient drop-out during the trial, which reduced the statistical power (Additional file 3: Supplementary Fig. S1b).

**Fig. 2:**
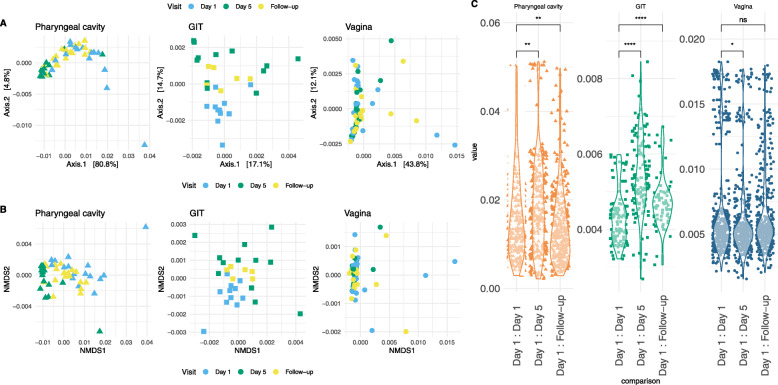
Beta diversity index of microbial communities using unweighted UniFrac distances with PCoA (**A**) or NMDS (**B**) projections on different body sites over time. (**C**) Violin plots showing the distribution of the first CCA scores for each visit and body type (* *P* value ≤0.05; ** *P* value ≤0.005; *** *P* value ≤0.0001 ns = nonsignificant, Wilcoxon test with Benjamini-Hochberg FDR correction)

The beta diversity index reflects differences between microbial communities across time-points or body sites. PCoA (Fig. 2a) and NMDS (Fig. 2b) of weighted UniFrac distances varied across the tested body sites with differences being less pronounced for Day 5 compared to Day 1 or Follow-up (Fig. 2c). This trend reflects the overall reduction in microbiome diversity at those body sites caused by gepotidacin up to Day 5 with a partial rebound of microbial communities by Follow-up, after cessation of dosing.

PERMANOVA (multivariate analysis with permutations) [14] confirmed that differences were significant (*P* value ≤0.001) between samples based on Visit, Type and Visit:Type variables. Constrained correspondence analysis (CCA), which explains the variability by selected variables, shows that microbial communities from different body sites were very distinct at Day 1 (Additional file 4 Supplementary Fig. S2). Those differences were reduced when patients received gepotidacin, then re-established following cessation of dosing. The greatest to least changes in microbiota community structure across time among the body sites were GIT, pharyngeal cavity and vagina, which is congruent with alpha diversity variation.

### Changes in detected abundances of bacterial genera

**Fig. 3:**
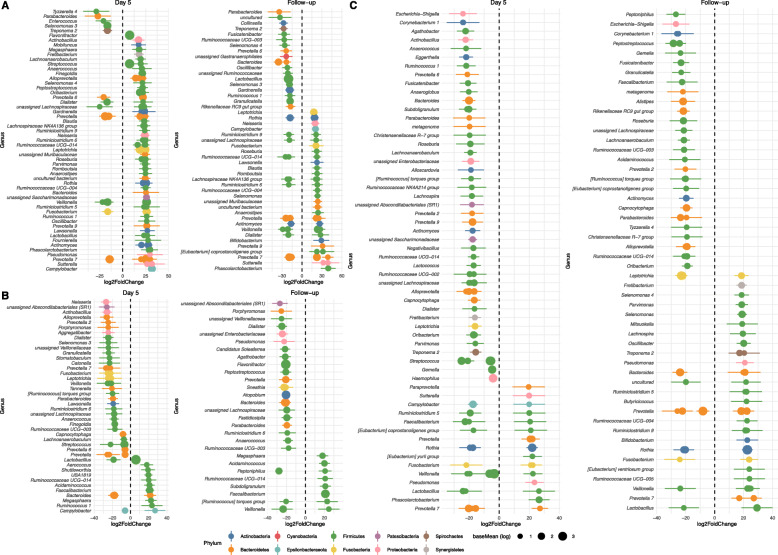
Changes in specific microbiota genera at Day 5 and Follow-up compared to Day 1 for the (**A**) gastro-intestinal tract (GIT); (**B**) pharyngeal cavity and (**C**) vagina. Size represents -log_10_ of FDR-adjusted *P*-value and lines represent CI at 95%

We evaluated changes in specific bacteria genera at Day 5 and Follow-up compared to Day 1 for each sampled body site. Among the three tested body sites, the GIT had the most variety of changed genera between Day 1 and Day 5. The greatest log-fold decreases in observed abundances were observed for the genera *Tyzzerella*, *Parabacteriodes*, *Enterococcus*, *Selenomonas*, *Treponema*, and several species of *Prevotella*, *Veillonella* and *Fusobacterium* (Fig. 3a). *Lactobacillus*, a core member of the GIT microbiota, rose in abundance. At Follow-up there was an increase in *Neisseria* spp. and a decrease in some core microbiota, such as *Lactobacillus*, compared to Day 1 which might be a consequence of the GIT microbiome returning to pre-dosing conditions. Other genera which showed the largest increases at Follow-up compated to Day 1 were *Phascolarctobacterium*, *Sutterella*, *Prevotella*, *Bifidobacterium*, *Dialister*, *Veillonella* and *Actinomyces* as well as members of the families Ruminococcaceae and Lachnospiraceae. There was no statistically significant change in the genus *Clostridioides* which includes the GIT pathogen *C. difficile*.

The pharyngeal cavity microbiome showed intermediate level changes at Day 5 and Follow-up relative to Day 1 (Fig. 3b). Genera negatively impacted at Day 5 and Follow-up include *Prevotella*, *Bacteriodes* and the oral specific genus *Porphyromonas*. The pharyngeal cavity microbiome also had a specific decrease of *Neisseria* at Day 5. Both Day 5 and Follow-up samples were characterized by increases of *Faecalibacterium* and *Ruminococcaceae* UCG-014.

The vaginal microbiome showed the least changes in diversity at Day 5 and Follow-up relative to Day 1 (Fig. 3c). However, at Day 5, significant reduction of the genus *Escherichia-Shigella* occurred, which is congruent with the antibacterial spectrum of gepotidacin, as well as minor reductions of the genus *Haemophilus* and the vaginal predominant genus *Lactococcus*. *Escherichia-Shigella* remained depleted at Follow-up vs Day 1 comparison, while *Lactococcus* was no longer significantly depleted, suggesting a recovery to Day 1 levels. The genus *Bacteriodes* showed depletion at Day 5 and an increase at Follow-up compared to Day 1.

### Changes in the detected abundances of specific bacterial pathogens

Next, we attempted to determine if in vitro susceptibility of specific pathogens to gepotidacin corresponded to the detectable abundances of similar genera in the microbiome as indirect evidence for in vivo drug effects. Previous in vitro studies reported minimum inhibitory concentrations (MIC) of gepotidacin needed to inhibit 90% of the bacteria tested as MIC_90_ ≤ 4 μg/mL [15, 16]. Tested species included the genera *Bacillus, Clostridioides, Escherichia-Shigella, Haemophilus, Neisseria, Staphylococcus* or *Streptococcus*.

**Fig. 4:**
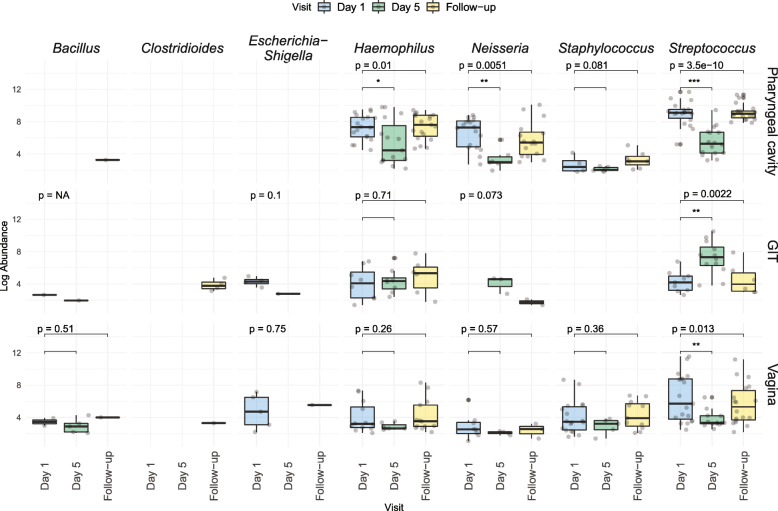
Overall trends in changes for specific pathogenic genera including *Bacillus*, *Clostridioides*, *Escherichia-Shigella*, *Haemophilus*, *Neisseria*, *Staphylococcus* and *Streptococcus*. (* *P* value ≤0.05; ** *P* value ≤0.005; *** *P* value ≤0.0001 ns = nonsignificant)

Overall trends suggested that gepotidacin in vitro MIC_90_ results are generally predictive of detectable abundances of these bacteria in the microbiome as measured clinically (Fig. 4). *Haemophilus* spp. and *Streptococcus* spp. were heavily impacted at Day 5 in the pharyngeal cavity and vagina but recovered at Follow-up. *Neisseria* spp., generally not found in the GIT microbiota, were reduced in the pharyngeal cavity and vagina at Day 5 and then rebounded at Follow-up. *Streptococcus* spp. showed transient increases only at Day 5 in the GIT. *Bacillus* spp. had low abundances across body sites and did not significantly change over time. Low abundances of *Clostridioides* spp. appeared in some Follow-up stool samples as discussed below.

We also looked at species-level changes in detected abundances for three specific pathogens, *Escherichia coli*, *Neisseria gonorrhoeae* and *Clostridioides difficile*, using phylogenetic analyses of actual 16S rRNA V4 sequences. For those terminal branches in the tree with sequences from our microbiome analyses, the log-transformed abundances were analyzed for each body site and time-point. A major caveat of this approach is that the average read length (~ 252 nucleotides) might be insufficient for robust taxonomic affiliation at the species or strain levels.

**Fig. 5:**
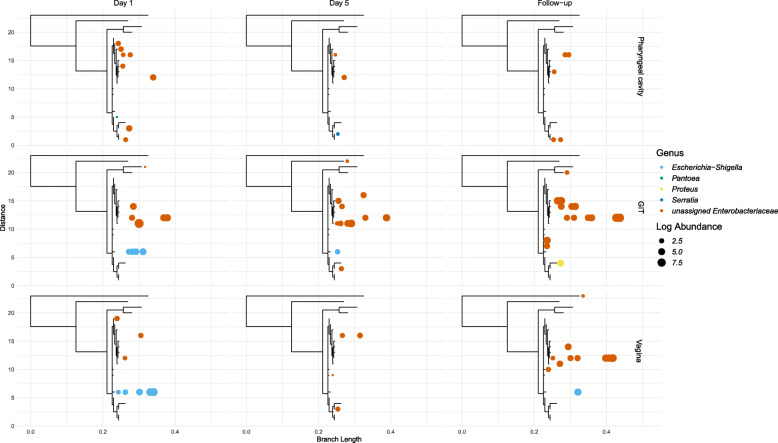
Species level changes in abundance for *E. coli* species using phylogenetic analyses of their 16S rRNA-V4 sequences and closely related sequences from the NCBI public database

Certain members of the *Enterobacterales* genus*,* which includes uropathogenic *E. coli* (UPEC), *Escherichia-Shigella* and other affiliated species, were detected across the three body sites at all time points (Fig. 5). While we could not resolve to the strain level using the available sequence data, *Escherichia-Shigella* reads were initially found at Day 1 in the GIT and vagina but greatly reduced or undetectable at Day 5 and Follow-up. The *Escherichia-Shigella* species cluster was not detected at any time points in the pharyngeal cavity, except for a minor occurrence of *Serratia* spp. at Day 5.

*N. gonorrhoeae* was detected at medium to high abundance at Day 1 in the pharyngeal cavity of a single patient (Additional file 5: Supplementary Fig. S3). The observed variation of *Neisseria* genus across the sampled body sites might be explained by changes of an unknown *Neisseria* spp. Finally, throughout our study, *C. difficile-*related sequences were only detected in low abundances in the GIT of four different patients at Follow-up (Additional file 6: Supplementary Fig. S4) but none of these patients presented clinically with infections caused by *C. difficile*.

## Discussion

In an exploratory analysis arm of a Phase 2a gepotidacin clinical trial in female subjects with uUTIs, we show the potential impact of gepotidacin on the microbiome at three different body sites across three time-points when patients were dosed orally at 1500 mg BID for 5 days. Significant declines in microbiome diversity relative to Day 1 were observed by Day 5 in microbial communities of the GIT, pharyngeal cavity and vagina as determined from DNA sequencing of 16S rRNA-V4 region amplicons from stool samples and pharyngeal and vaginal swabs, respectively. The three microbial communities showed significant recovery in diversity at Follow-up (collected at 28 ± 3 days). Our analyses suggest that the overall effects of gepotidacin on these body sites’ microbiome are relatively transient and reversible.

Although gepotidacin was dosed orally and, therefore, expected to mainly affect the GIT microbiome, we also observed changes in the distal pharyngeal and vaginal microbiomes. The overall magnitude of changes in microbiome alpha diversity were greatest for the pharyngeal cavity, followed by GIT. Vaginal microbiome diversity did not significantly change although some pathogenic genera including *Haemophilus* and *Streptococcus*, remained depleted at Follow-up. During the same Ph2a clinical trial, localized concentrations of gepotidacin were measured using swab samples collected over a 4 day period which revealed the rank-order of body-sites, in terms of high-to-low drug concentrations, as rectal > vaginal > pharyngeal [12]. The vaginal microbiome might be less affected by gepotidacin due to its naturally lower diversity skewed towards Gram-positive species such as lactic-acid producing *Lactobacillus* spp. [17]. In vitro susceptibility testing of nine different *Lactobacillus* spp. suggested variable susceptibility to gepotidacin (MIC range ≤ 0.015–2 μg/mL) (unpublished data). Collectively, these findings suggest that differences in microbiota dynamics at these three specific body sites might be due to multiple factors including localized drug concentrations and the intrinsic composition of the microbiome at those locations. Additional in vitro studies involving individual microbiota species and strains are necessary to understand the lineage specific factors impacting susceptibility to gepotidacin and other antibiotics.

Analyses of impacted microbiota genera suggest that gepotidacin has a distinct and selective in vivo spectrum of activity which might partially explain the observed rebound of the microbial community post-treatment. Our study suggests that gepotidacin potentially affects the abundance of several genera, including those of known pathogens, the *Bacillus, Escherichia, Clostridioides, Haemophilus, Neisseria, Staphylococcus,* and *Streptococcus*. Although *C. difficile* reads could be detected at low concentration in stool samples of four patients at Follow-up, these were not statistically significant with respect to other time points or body sites and no patient in the study presented with clinically relevant *C. difficile* infections. Gepotidacin did affect the abundance of some genera considered part of the core healthy microbiome, such as *Prevotella* spp. However, other proposed beneficial taxa, including *Lactococcus* and *Lactobacillus*, were mostly unchanged. Utilization of qPCR and metagenomics assays would have provided higher resolution of antibiotic effects at the species and strain level.

Several studies have reported the effects of fluoroquinolones on the GIT microbiome. Fluoroquinolones also target bacterial DNA gyrase and topoisomerase IV, although gepotidacin has distinct molecular interactions with these proteins which avoid known amino acids associated with bacterial resistance to fluoroquinolones [1]. Dethlefsen and Relman measured microbiota changes using 16S rRNA amplicons in three healthy individuals given two courses of the fluoroquinolone ciprofloxacin over a period of 10 months [18]. They observed a rapid loss of diversity within 3–4 days of initial dosing. The microbiome began to recover within 1 week after last dose although not to the baseline, pre-dose situation. Willmann et al. performed a longitudinal, microbiome metagenomics analyses of two hospital patient cohorts treated prophylactically with the antibiotics cotrimoxazole or ciprofloxacin [19]. At the end of the observation period, 6 days post-last dose, the ciprofloxacin cohort showed a significantly greater reduction in microbiota species diversity and richness compared to the cotrimoxazole cohort. However, multi-variate analyses which integrated various clinical measures, suggested that higher dosing of ciprofloxacin relative to cotrimoxazole, nearly four-fold greater, might be a key contributing factor. Focusing solely on the enterococci, de Lastours et al. found that stool samples from healthy volunteers had an increase in *Enterococcus* species with fluoroquinolone resistant loci relative to baseline 42 days after a 2 week dosing regimen of ciprofloxacin [20]. As for other antibiotic classes, Cannon et al. [21] compared the effects of vancomycin and surotomycin, a bactericidal cyclic lipopeptide, on the microbiome in a Phase 2 trial against *C. difficile* infections. Using qPCR, they assessed changes among 10 microbiota species in fecal samples collected from 8 control patients dosed orally with 125 mg of vancomycin or surotomycin for 10 days at 7 timepoints (3 pre-dose and dosed and 4 follow-up samplings). Vancomycin-treated patients were found to have notable suppression of microbiota abundances at 42 days compared to surotomycin, with *Veillonella* spp.*, Bacteriodes* spp. and *Prevotella* spp. failing to recover to baseline values. In comparison to fluoroquinolones, the microbiome seems to recover to near pre-dose status within a few weeks post-dosing of gepotidacin.

Chng et al. [11] recently published a meta-analysis of more than 500 microbiome profiles from 117 individuals across four different continents receiving antibiotic therapy. They identified 21 bacterial species associated with post-treatment GIT microbiome recovery which included members of the genera *Bacteroides, Bifidobacterium* and *Ruminococcus*, also seen elevated in our Follow-up stool samples. However, several genera differed between the two studies which might be attributed dissimilarities in administered drug classes and study subject cohorts. In Chng et al. cohorts, most patients received antiobiotics from amoxicillin-clavulanic acid, lincosamides or macrolides classes while the sole fluroquinolone, ciprofloxacin, was administered to only a few healthy volunteers. There was no sub-cohort analysis of microbiota changes by drug class. Thus further clinical studies involving head-to-head comparisons of antibiotic induced microbiome changes in patients would be useful in elucidating the specific effects of different compound classes on human microbiota.

One caveat of our study is that all subjects had a bacterial infection at enrollment thus their baseline microbiome composition might be affected by pathogen-induced dysbiosis. Therefore, we have no information about the impact of gepotidacin on re-establishing a healthy microflora. However, since clearing of the infection was confirmed, the remaining differences in microbiota composition between Day 1 and Follow-up might hint of reversion to a non-dysbiotic state at the sampled body sites. A second caveat of our study is that the microbiome data reflects changes in the detectable abundances of microbiota taxa and does include measures of absolute bacterial load. We did show that previously determined in vitro susceptibility of specific pathogens to gepotidacin corresponded to the abundances of similar genera in the microbiome as indirect evidence for in vivo drug effects. While direct and more specific measures of bacterial abundance, for example using qPCR of universal bacterial specific loci might be interesting, their interpretation in the context of microbiome could be complicated by rapid niche-filling by microbiota with low susceptibility to gepotidacin which could result in sustained levels of overall bacterial load across time-points. A third caveat is that without metagenomic data, we are unable to assess the potential impact of gepotidacin on the occurrences of specific drug resistance gene loci, the so-called resistome, in the microbiota from GIT, pharyngeal or vaginal samples.

## Conclusion

Our study provides evidence of relatively rapid microbiome recovery at multiple body sites for patients with uUTIs being treated with the novel antibiotic, gepotidacin. The clinical ramifications of facilitating microbiome rebound after antibiotic treatment needs further investigation using larger patient cohorts as well as multiple comparisons across different antibiotic regimens.

## Methods

### Study population and sample collection

The microbiome analysis was an exploratory endpoint in a Phase 2A clinical trial (ClinicalTrials.gov: NCT03568942). Study design, protocols and primary findings for this trial were previously published [12]. Eligible female subjects received twice daily (BID) dose of gepotidacin 1500 mg (mg) for 5 days via oral route. The total duration of the study was approximately 28 days. This trial is compliant with CONSORT guidelines. Samples for microbiome analyses were collected from 22 subjects with informed consent in accordance with study protocols at three time-points: Day 1 (pre-dose); Day 5 (end of dosing or post dose) and; Follow-up (visit around Day 28 ± 3 days). Three different body sites were sampled, namely gastro-intestinal tract (GIT; stool sample), pharyngeal cavity (saliva sterile swab) and vaginal (vaginal sterile swab).

### DNA extraction and sequencing

All samples were stored at − 80 °C prior to DNA extraction and sequencing. For quality control purposes, all genomic extractions, sequencing and data analyses were performed in a single, central next generation sequencing (NGS) laboratory of GlaxoSmithKline Research and Development (GSK R&D) in Collegeville, Pennsylvania, USA.

Genomic DNA was isolated from stool samples using QIAamp PowerFecal DNA Kit (Qiagen, Hilden, Germany) according to the manufacturer’s instructions. Genomic DNA was isolated from saliva and vaginal samples by first concentrating the preservation solution using ultra centrifugal filters (Amicon, EMD Millipore, Darmstadt, Germany) followed by using QIAamp PowerSoil Pro Kit (Qiagen, Hilden, Germany) according to manufacturer’s instructions. Each genomic DNA sample was quantified by Qubit fluorometric kit (Invitrogen, Thermofisher, Waltham, MA). PCR amplification of the 16S rRNA V4 region was conducted with primers, 515f (5′- GTGCCAGCMGCCGCGGTAA-3′) and 806r (5′-GGACTACHVGGGTWTCTAAT-3′) including an 8-nt index sequence, a 10-nt pad sequence, a 2-nt linker, and the appropriate Illumina adapter [22, 23]. The index sequences were selected to be at least 2-nt different from all other indices in use, and when combined, they provide an equal intensity in the two light channels used by the sequencer (i.e., green channel [G/T] and red channel [A/C]) [24]. Each 25 μL PCR reaction containing on average 100 ng of genomic DNA, KAPA HiFi HotStart ReadyMix (KAPABIOSYSTEMS, Wilmington, MA), and 0.2 μM of each primer (Integrated DNA Technologies, Coralville, IA). PCR was performed on an ABI 9700 thermocycler and included the following cycling steps: Initial denaturing at 95 °C for 3 min followed by 35 cycles of 95 °C × 20 s, 60 °C × 15 s, and 72 °C × 30 s ending with a 72 °C 1 × minute extension. A 2ul aliquot of each resulting amplicon was run on a 2.0% E-gel 96 SYBR Safe Stain Precast agarose gel (Invitrogen, Waltham, MA) to check quality and quantity. Positive (ZymoBIOMICS Community and DNA Standards) and negative controls consisting of reagent-only isolation kit reactions along with no-template amplifications using Microbial DNA-Free Water (Qiagen, Hilden, Germany) were included for all isolation steps, PCR reactions and DNA sequencing runs. All PCR products were verified using both Qubit quantitation and gel electrophoresis for sensitive resolution of the amplicon of interest. All negative controls were shown to be free of DNA contamination by a combination of negative Qubit High Sensitivity results and a lack of detectable E-gel amplicon bands.

Amplicons were then purified using a magnetic bead capture kit (Ampure XP; Agencourt) and quantified using a fluorometric kit (Qubit; Invitrogen). The purified amplicons were then pooled in equimolar concentrations using a SequalPrep plate normalization kit (Invitrogen), and the final concentration of the library was determined using a SYBR green quantitative PCR (qPCR) assay with primers specific to the Illumina adapters (KAPABIOSYSTEMS, Wilmington, MA).

To check for proper cluster density and sample normalization, an Illumina MiSeq single-end 26 bp + 8 bp dual index sequencing run was performed using the MiSeq instrument. Unique dual-index barcodes were designed per Illumina recommendation to avoid sequencing artifacts due to index hopping. The pool was mixed with a PhiX library (Illumina, San Diego CA) at a ratio of .5:9.5 in order to increase the entropy of the library. A final MiSeq 2 × 150 bp + 8 bp dual index sequencing run was performed on the pooled samples.

Reads were first filtered to remove the PhiX library reads. All reads mapping to the Enterobacteria phage PhiX 174 reference genome (GenBank: NC_001422.1) using the software Bowtie (v1.0.1) [25] were removed from the analysis. The paired reads were next merged with the software PEAR (v0.9.5) [26]. DNA sequence data are available from the National Centre for Biotechnology Information Sequence Read Archive (SRA) under BioProject ID: PRJNA630295 and SRA submission: SUB7386163.

### Data analysis

All statistical analyses were post-hoc (i.e. defined after unblinding the clinical study). Reads from 16S rRNA-V4 regions (≥19 M total) were analyzed using Qiime2, v2018.8 [27]. Samples from positive and negative control wells were analyzed separately. All 16S rRNA-V4 reads were trimmed where average quality score dropped below 25 (150 and 149 base pairs [bp] for forward and reverse reads, respectively) and dereplicated using DADA2 [28] with paired-end default settings (including quality control, trimming, pair-joining and chimera removals) resulting in 90.74% retained reads. The 16S rRNA-V4 representative amplicon sequence variants (ASVs) were assigned to the SILVA 132 database [29] by using a multinomial naïve Bayes classifier [30]. Phylogenetic trees were built in Qiime2 using MAFFT [31] and fasttree [32]. Finally, data were exported from Qiime2 and converted into the BIOM v1.5 format [33] for easier import into R.

Diversity analyses were performed using the R packages “Phyloseq” v1.34.0 [34] and “vegan” v2.5–7 [35], “microbiome” 1.12–0 [36] and “picante” 1.8.2 [37]. Alpha diversity was calculated using Berger-Parker, Faith’s PD, observed ASVs, Simpson’s and Shannon’s diversity indexes on non-normalized data [38]. Beta diversity analysis was performed on log-normalized data to minimize biases related to rarefaction [39] and included principal coordinate analysis (PCoA), non-metric multidimensional scaling (NMDS) and Constrained Correspondence Analysis (CCA), performed on either Bray-Curtis or unweighted UniFrac distances [40]. PERMANOVA was used to test for significance with default setting of 999 permutations. Differential abundance tests were performed on non-normalized ASVs pseudocounts with the DESeq2 package (parametric model, Wald’s test) [41]. Per-body-site contrasts were made for each time point against Day 1 (*P* ≤ 0.01%). The false discovery rate (FDR) method was used to adjust *P*-values for multiple tests where applicable [42].

## Supplementary Information

**Additional file 1: Supplementary Table S1.** Summary of samples collected and passing quality control (QC) for subsequent analyses.

**Additional file 2: Supplementary Data File S1:** Relative abundances of assigned bacterial taxa for each body site and time-point.

**Additional file 3: Supplementary Figure S1.** Changes in microbiota community as measured by different indices of alpha diversity (A) and pairwise statistical analysis of alpha diversity values for the subset of patients having the full set of three visit throughout the trial (*n* = 16) (B). The initial, lower placed value in each comparison is from the overall ANOVA (* *P* value ≤0.05; ** *P* value ≤0.005, ns = nonsignificant).

**Additional file 4: Supplementary Figure S2.** Multivariate analyses of changes in microbial community using constrained correspondence analysis (CCA) on Bray-Curtis distances showing recovery at Follow-up (A) and violin plots showing the distribution of the first CCA scores for each visit and body type (B) (* *P* value ≤0.05; ** *P* value ≤0.005; *** *P* value ≤0.0001 ns = nonsignificant, Wilcoxon test with Benjamini-Hochberg FDR correction).

**Additional file 5: Supplementary Figure S3.** Species-level changes in abundance for *Neisseria gonorrhoeae* related species using phylogenetic analyses of their 16S rRNA gene -V4 sequences as well as related sequences from NCBI public database.

**Additional file 6: Supplementary Figure S4.** Species-level changes in abundance for *Clostridioides* species using phylogenetic analyses of their 16S rRNA-V4 sequences as well as related sequences from NCBI public database. Occurrence of *C. difficile* in the GIT samples from four subjects at Follow-up is labeled.

## Data Availability

DNA sequence data and metadata are available from the National Centre for Biotechnology Information Sequence Read Archive (SRA) under BioProject ID: PRJNA630295]. Metadata are formatted following the MIMARKS standard and included in the BioProject repository. Additional files are available as Supplementary Electronic Material. Code to reproduce the analysis is available at https://github.com/andreanuzzo/gepotidacin_phIIa_microbiome. This study is registered at clinicaltrials.gov with the identifier NCT03568942. Anonymized individual participant data and study documents can be requested for further research from www.clinicalstudydatarequest.com.

## Abbreviations

uUTI: Uncomplicated urinary tract infection
GIT: Gastrointestinal tract
MIC: Minimum inhibitory concentrations
BID: Two times a day dosing
