# Correction to: Microbiome recovery in adult females with uncomplicated urinary tract infections in a randomised phase 2A trial of the novel antibiotic gepotidacin (GSK2140944)

**DOI:** 10.1186/s12866-021-02356-2

**Published:** 2021-10-26

**Authors:** Andrea Nuzzo, Stephanie Van Horn, Christopher Traini, Caroline R. Perry, Etienne F. Dumont, Nicole E. Scangarella-Oman, David F. Gardiner, James R. Brown

**Affiliations:** 1 Human Genetics, GlaxoSmithKline R&D, Medicines Research Centre, Gunnels Wood Road, Stevenage, SG1 2NY UK; 2 Functional Genomics, GlaxoSmithKline R&D, Collegeville, PA USA; 3 Development, GlaxoSmithKline R&D, Collegeville, PA USA; 4 Medicines Opportunities Research Unit, GlaxoSmithKline R&D, Collegeville, PA USA; 5 Human Genetics, GlaxoSmithKline R&D, Collegeville, PA USA; 6 Present Address: Kaleido Biosciences, 65 Hayden Avenue, Lexington, MA 02421 USA

**Correction to:** ***BMC Microbiol*** **21, 181 (2021)**

10.1186/s12866-021-02245-8

Following the publication of the original article [1], the authors would need to modify the typographical error in the compound number in article title, from GSK140944 (current) to GSK2140944 (correct). The correct article title is shown above.

The original article has been corrected.
