# Supplementary material for: Microbiome recovery in adult females with uncomplicated urinary tract infections in a randomised phase 2A trial of the novel antibiotic gepotidacin (GSK140944)
Source: BMC Microbiol. 2021 Jun 15;21:181. doi: 10.1186/s12866-021-02245-8 (PMC8207760; doi:10.1186/s12866-021-02245-8)
Supplement: Supplementary file 1 — Additional file 1: Supplementary Table S1. Summary of samples collected and passing quality control (QC) for subsequent analyses. [file 12866_2021_2245_MOESM1_ESM.docx]

**Supplementary Table S1.** Summary of samples collected and passing quality control (QC) for subsequent analyses. All study subjects were female.

| Body site | Day 1 | | Day 5 | | Follow-up | | Total Collected | Total Pass QC |
| --- | --- | --- | --- | --- | --- | --- | --- | --- |
|  | Collected | Passed QC | Collected | Passed QC | Collected | Passed QC |  |  |
| GI Tract  (stool) | 13 | 11 | 13 | 12 | 10 | 6 | 36 | 29 |
| Pharyngeal  (saliva) | 21 | 18 | 20 | 16 | 19 | 19 | 60 | 53 |
| Vaginal  (swabs) | 21 | 21 | 20 | 19 | 19 | 19 | 60 | 59 |
| **Total** | **55** | **50** | **53** | **47** | **48** | **44** | **156** | **141** |
