# Supplementary figures and images for: Microbiome recovery in adult females with uncomplicated urinary tract infections in a randomised phase 2A trial of the novel antibiotic gepotidacin (GSK140944)
Source: BMC Microbiol. 2021 Jun 15;21:181. doi: 10.1186/s12866-021-02245-8 (PMC8207760; doi:10.1186/s12866-021-02245-8)

shape ▲ Pharyngeal cavity ■ GIT ● Vagina Visit Day 1 Day 5 Follow-up

**A**

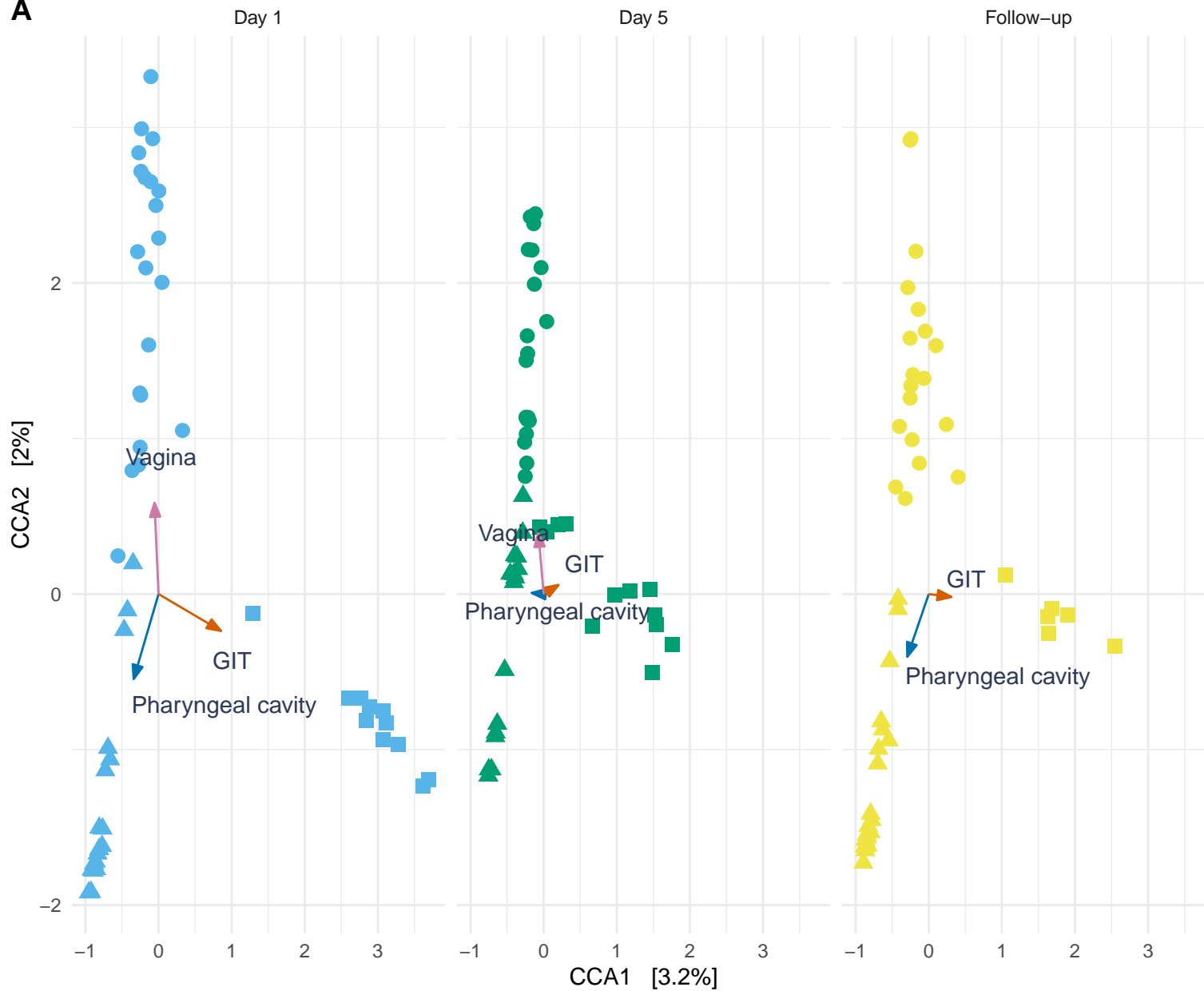

**B**

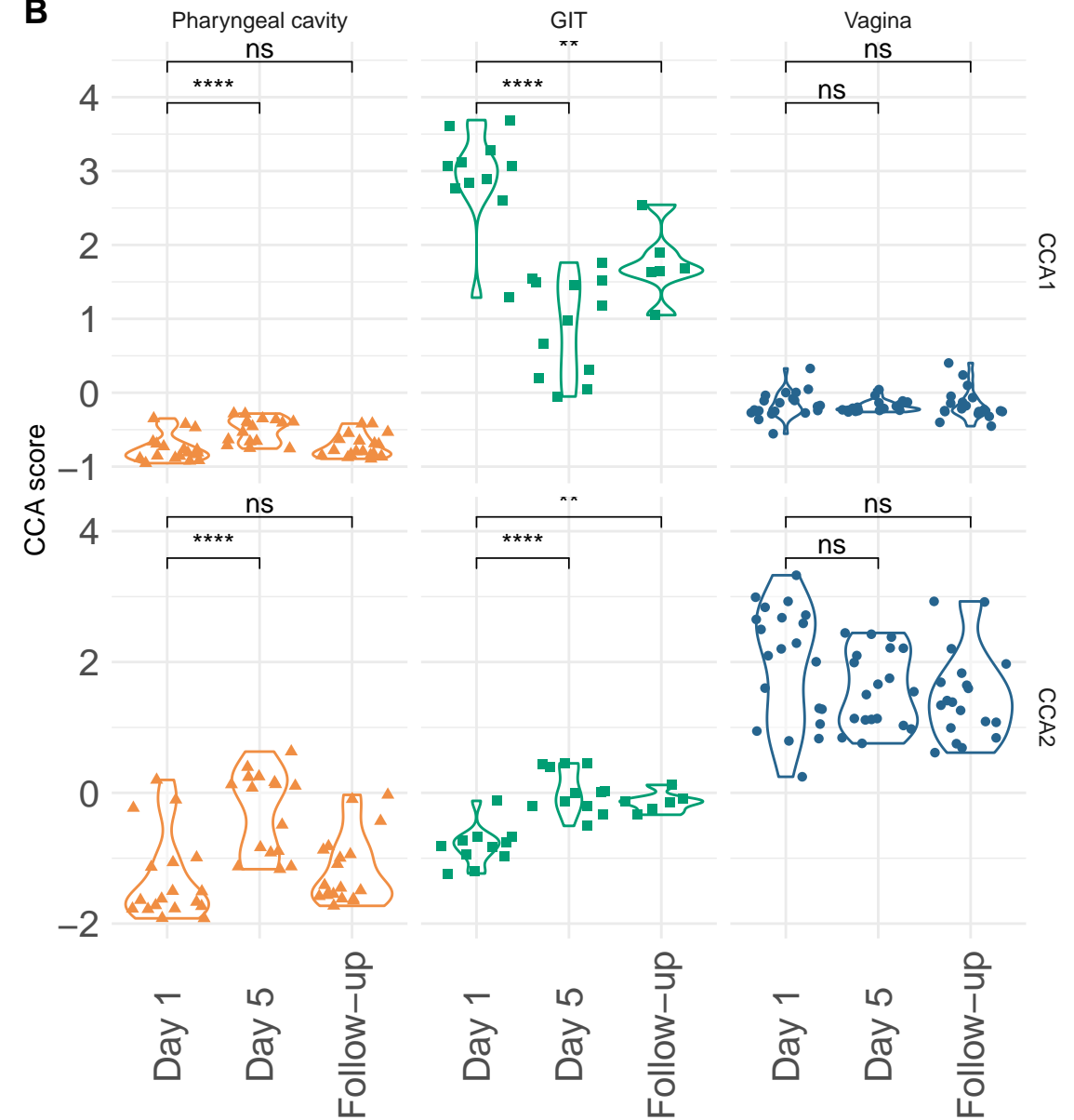

Supplement: Supplementary file 4 — Additional file 4: Supplementary Figure S2. Multivariate analyses of changes in microbial community using constrained correspondence analysis (CCA) on Bray-Curtis distances showing recovery at Follow-up (A) and violin plots showing the distribution of the first CCA scores for each visit and body type (B) (* P value ≤0.05; ** P value ≤0.005; *** P value ≤0.0001 ns = nonsignificant, Wilcoxon test with Benjamini-Hochberg FDR correction). [file 12866_2021_2245_MOESM4_ESM.pdf]

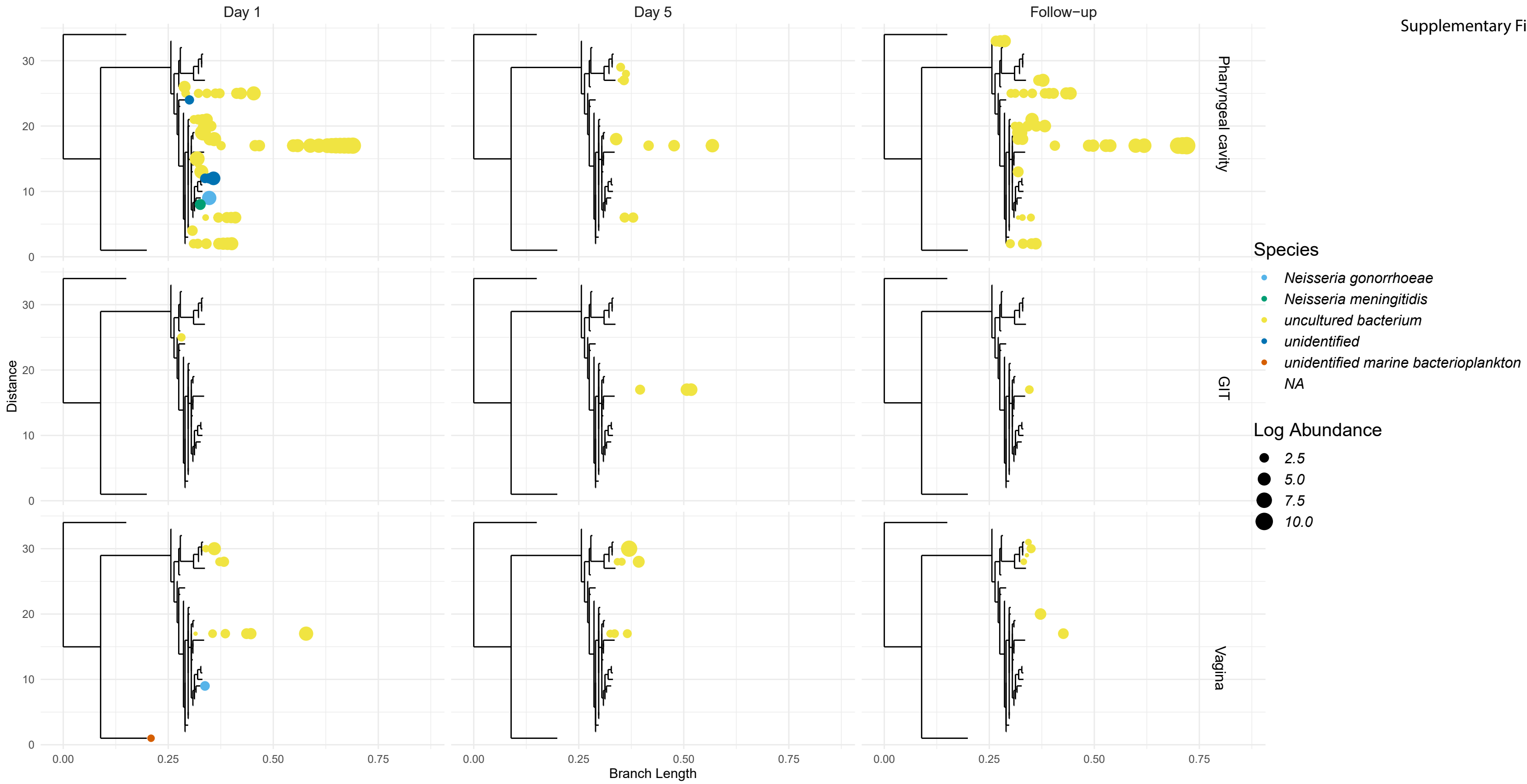

Supplement: Supplementary file 5 — Additional file 5: Supplementary Figure S3. Species-level changes in abundance for Neisseria gonorrhoeae related species using phylogenetic analyses of their 16S rRNA gene -V4 sequences as well as related sequences from NCBI public database. [file 12866_2021_2245_MOESM5_ESM.pdf]

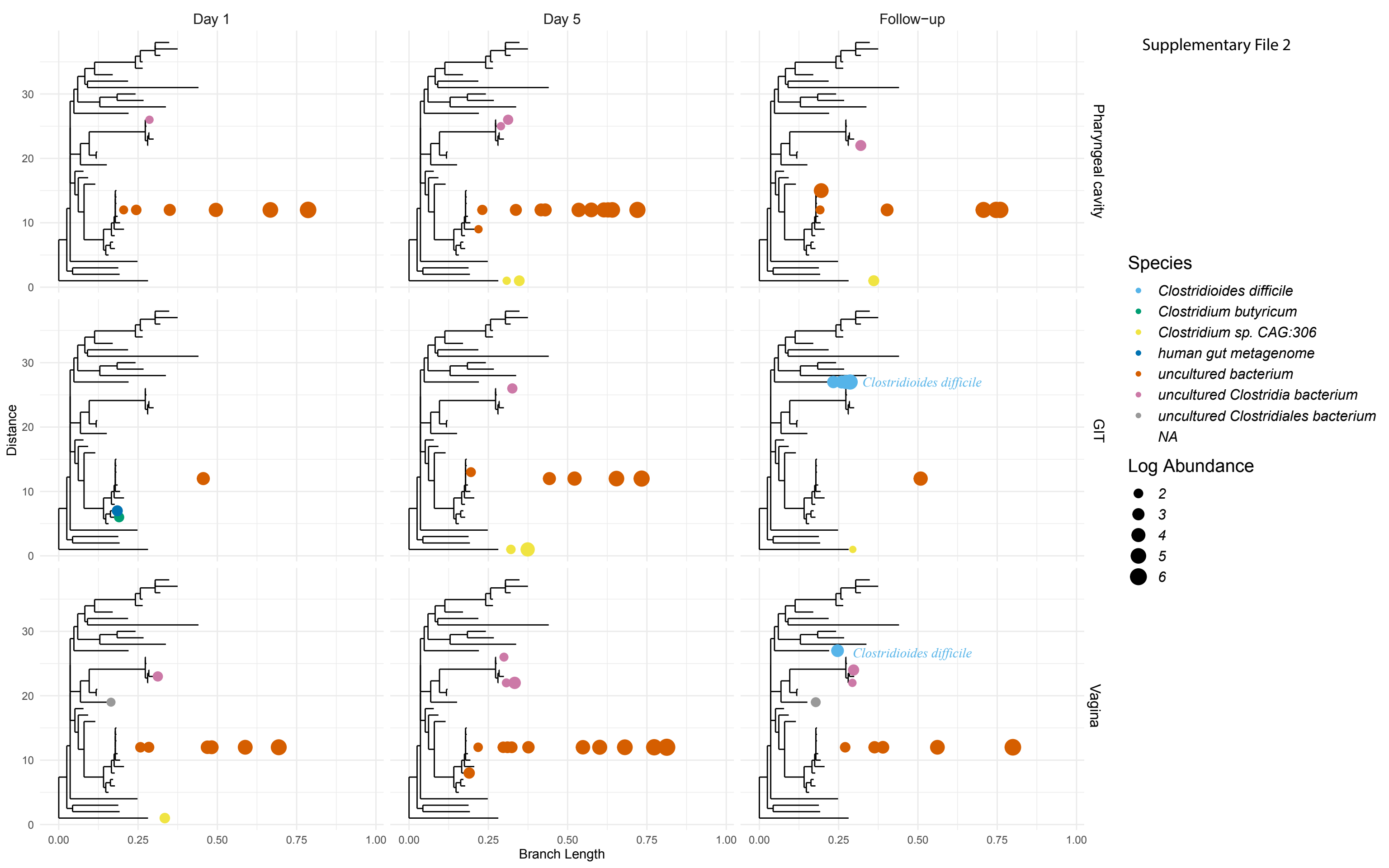

Supplement: Supplementary file 6 — Additional file 6: Supplementary Figure S4. Species-level changes in abundance for Clostridioides species using phylogenetic analyses of their 16S rRNA-V4 sequences as well as related sequences from NCBI public database. Occurrence of C. difficile in the GIT samples from four subjects at Follow-up is labeled. [file 12866_2021_2245_MOESM6_ESM.pdf]
