# Supplementary material for: Microbiome recovery in adult females with uncomplicated urinary tract infections in a randomised phase 2A trial of the novel antibiotic gepotidacin (GSK140944)
Source: BMC Microbiol. 2021 Jun 15;21:181. doi: 10.1186/s12866-021-02245-8 (PMC8207760; doi:10.1186/s12866-021-02245-8)
Supplement: Supplementary file 3 — Additional file 3: Supplementary Figure S1. Changes in microbiota community as measured by different indices of alpha diversity (A) and pairwise statistical analysis of alpha diversity values for the subset of patients having the full set of three visit throughout the trial (n = 16) (B). The initial, lower placed value in each comparison is from the overall ANOVA (* P value ≤0.05; ** P value ≤0.005, ns = nonsignificant). [file 12866_2021_2245_MOESM3_ESM.pdf]

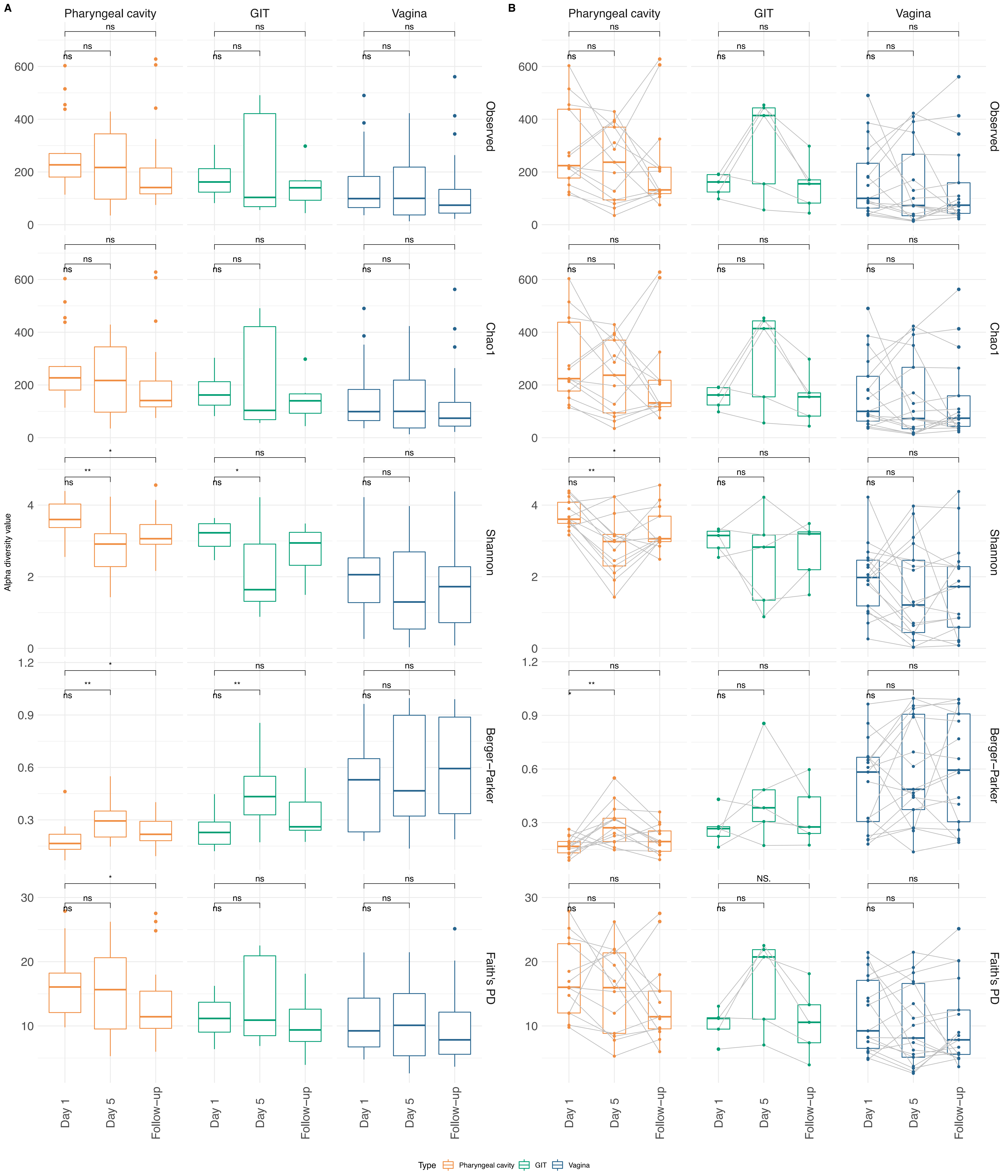
A
B
Pharyngeal cavity
GIT
Vagina
Observed
Chao1
Shannon
Berger–Parker
Faith's PD
Alpha diversity value
Day 1
Day 5
Follow–up
Type
Pharyngeal cavity
GIT
Vagina
